# Trait Interindividual Differences in the Magnitude of Subjective Sleepiness from Sleep Inertia

**DOI:** 10.3390/clockssleep3020019

**Published:** 2021-06-03

**Authors:** Kirsie R. Lundholm, Kimberly A. Honn, Lillian Skeiky, Rachael A. Muck, Hans P. A. Van Dongen

**Affiliations:** Sleep and Performance Research Center & Elson S. Floyd College of Medicine, Washington State University, Spokane, WA 99202, USA; kirsie.lundholm@wsu.edu (K.R.L.); kimberly.honn@wsu.edu (K.A.H.); lillian.skeiky@wsu.edu (L.S.); rachael.muck@wsu.edu (R.A.M.)

**Keywords:** human phenotype, intraclass correlation coefficient, Karolinska Sleepiness Scale, nonlinear mixed-effects modeling, on-call work, recovery sleep, sleep extension, sleep restriction

## Abstract

In shift work settings and on-call operations, workers may be at risk of sleep inertia when called to action immediately after awakening from sleep. However, individuals may differ substantially in their susceptibility to sleep inertia. We investigated this using data from a laboratory study in which 20 healthy young adults were each exposed to 36 h of total sleep deprivation, preceded by a baseline sleep period and followed by a recovery sleep period, on three separate occasions. In the week prior to each laboratory session and on the corresponding baseline night in the laboratory, participants either extended their sleep period to 12 h/day or restricted it to 6 h/day. During periods of wakefulness in the laboratory, starting right after scheduled awakening, participants completed neurobehavioral tests every 2 h. Testing included the Karolinska Sleepiness Scale to measure subjective sleepiness, for which the data were analyzed with nonlinear mixed-effects regression to quantify sleep inertia. This revealed considerable interindividual differences in the magnitude of sleep inertia, which were highly stable within individuals after both baseline and recovery sleep periods, regardless of study condition. Our results demonstrate that interindividual differences in subjective sleepiness due to sleep inertia are substantial and constitute a trait.

## 1. Introduction

Shift work is often associated with sleep loss and/or sleep displacement (i.e., circadian misalignment), leading to physiological, behavioral, and subjective sleepiness [1,2]. There are large, systematic differences in the impact of shift schedules on individuals and in the level of sleepiness they experience [3,4]. In settings where workers may be called upon immediately after being awakened from sleep—e.g., on-call operations [5], emergency response [6], healthcare [7], and split duty schedules [8]—individuals are at further risk of sleepiness and potentially making sleepiness-related errors, due to sleep inertia (SI). This refers to the transient period of sleepiness, grogginess, disorientation, and decreased cognitive performance that occurs upon awakening [9]. Although there is some evidence people with different chronotypes (“early birds” and “night owls”) may differ in the extent to which they experience SI [10], almost nothing is known about the scale of systematic interindividual differences in susceptibility to SI.

The literature on SI has been reviewed elsewhere [11,12,13]. Briefly, SI is greatest right after waking up, with the effect dissipating exponentially and essentially disappearing within approximately an hour following awakening [14,15]. The magnitude of SI depends on sleep/wake history and circadian timing [16,17], such that SI causes more profound sleepiness after prior sleep restriction [18,19], during the circadian nadir [16,20], and when awakening from deep (non-REM) sleep [21,22]. Caffeine, an adenosine antagonist, suppresses SI [23,24], and there is theoretical evidence that SI is an adenosine-mediated phenomenon closely related to sleep/wake regulation [25].

Given large, trait interindividual differences in other manifestations of sleep/wake regulation—such as habitual sleep duration [26], sleep architecture [27], and vulnerability to the sleepiness-inducing effects of sleep deprivation [28]—we hypothesized that there are also large, trait interindividual differences in the magnitude of SI. To investigate this, we made use of data from a previously published study [28] in which trait interindividual differences in neurobehavioral deficits caused by sleep deprivation were evaluated. In this laboratory study, participants were each exposed to 36 h of total sleep deprivation, preceded by a baseline sleep period and followed by a recovery sleep period, on three separate occasions. In the week prior to each laboratory session and on the baseline night in the laboratory, participants were required to either extend their time in bed to 12 h per day (prior sleep extension condition) or restrict their sleep to 6 h per day (prior sleep restriction condition). While in the laboratory, participants underwent neurobehavioral testing every 2 h while awake, starting right after scheduled awakening. Testing included self-reporting of subjective sleepiness on the Karolinska Sleepiness Scale (KSS) [29].

Here, to investigate trait interindividual differences in subjective sleepiness in the magnitude of SI, we used the KSS data collected after baseline sleep, following prior sleep extension as well as prior sleep restriction, and after recovery sleep. In line with previously established criteria to assess trait interindividual differences [27,28], we set out to determine whether sleepiness due to SI was (a) substantially different between individuals; (b) highly stable within individuals; and (c) robust to experimental manipulation of sleep/wake history. We employed the intraclass correlation coefficient (ICC), which expresses systematic variability between individuals relative to overall variability in the data set [30], to quantify the extent to which individual susceptibility to SI is a trait.

## 2. Materials and Methods

### 2.1. Participants

Twenty-one healthy young adults completed the study. One participant gave identical responses for self-reported sleepiness throughout the study. As we could not be certain these responses were truthful and because they could inflate estimates of trait variability, data from this participant were not used. As such, data were available for *N* = 20 participants, 12 men and 8 women, ranging in age from 21 to 38 years (mean ± SD: 29.3 ± 5.7 years). All participants were physically and psychologically healthy, with no sleep or circadian disorders and free of medications (excluding oral contraceptives) and drugs. They reported good habitual sleep, between 6.5 and 8.5 h in duration and getting up between 06:30 and 08:30, and were neither extreme morning nor extreme evening types, as assessed with the Composite Scale of Morningness [31]. The study was approved by the Institutional Review Board of the University of Pennsylvania, and all participants gave written informed consent.

### 2.2. Experimental Design

The study was conducted in the General Clinical Research Center of the Hospital of the University of Pennsylvania, in a controlled laboratory environment with dim light (less than 50 lux) and fixed ambient temperature (21 ± 1 °C). Participants first underwent a laboratory adaptation session to practice the neurobehavioral test battery (described below) and acclimate to sleeping in the laboratory while being recorded polysomnographically. Participants then came to the laboratory three more times, at intervals of at least 2 weeks. Each of these three sessions involved a baseline sleep opportunity, a 36-h period of total sleep deprivation, and a recovery sleep opportunity. In randomized order, one of the three laboratory sessions was preceded by a requirement to restrict time in bed at home to 6 h per day for a week (prior sleep restriction condition, PSR). The other two laboratory sessions were preceded by a requirement to extend time in bed at home to 12 h per day for a week (prior sleep extension condition, PSE). Adherence to these requirements was verified by means of wrist actigraphy and a sleep/wake diary, and participants called a time-stamped telephone recorder every morning and every night to report their bedtimes [28]. In the PSR condition, the laboratory baseline sleep opportunity was also restricted to 6 h; in the PSE condition, the laboratory baseline sleep opportunities were 12 h. The recovery sleep opportunity was 12 h in all cases. All laboratory sleep periods were recorded with digital polysomnography (Vitaport 3; TEMEC Instruments, Kerkrade, The Netherlands) and scored using criteria set forth by Rechtschaffen and Kales [32]. 

For each of the three laboratory sessions, participants entered the laboratory at 15:00 on the first of 4 consecutive days. On that first day, they practiced the neurobehavioral test battery again. In the PSE condition, they then went to bed with the lights being turned off at 22:00 for a 12-h baseline sleep period. In the PSR condition, they performed the neurobehavioral test battery at 22:00, 00:00 (midnight), and 02:00, and then went to bed with the lights being turned off at 04:00 for a 6-h sleep period. The 36-h sleep deprivation period began at 10:00 on the second day. Participants were awakened by turning on the lights and verbally prompted to begin neurobehavioral testing right away. The neurobehavioral test battery was repeated at 2-h intervals throughout the sleep deprivation period, during which wakefulness was monitored continuously by trained staff. On the third day, participants went to bed with the lights being turned off at 22:00 for a 12-h recovery sleep period. On the fourth day, participants were again awakened at 10:00 by turning on the lights and verbally prompted to begin neurobehavioral testing right away. Participants went home at around 12:00 that day.

Throughout the study, participants completed the neurobehavioral test bouts while seated at a desktop computer. Each test bout began with a computerized version of the KSS, a Likert-type self-report measure of subjective sleepiness with scores ranging from 1 to 9 [33]. Anchoring was provided at the odd scores: 1 = very alert; 3 = alert, normal level; 5 = neither alert nor sleepy; 7 = sleepy, but no effort to keep awake; 9 = very sleepy, great effort to keep awake, fighting sleep. This first KSS (KSS-1) was followed by two other subjective assessments and a variety of performance tests described previously [28], which took approximately 60 min to complete. A second KSS (KSS-2) was included toward the very end of the test bout, 58.2 ± 0.2 min (mean ± SD) after the first.

**Figure 1:**
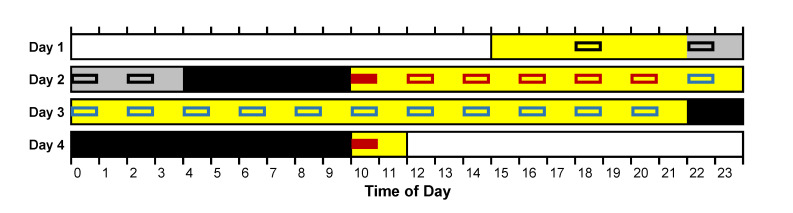
Schematic of the laboratory sessions. Yellow segments indicate scheduled wakefulness with neurobehavioral testing; black segments indicate scheduled sleep; and the grey segment indicates scheduled sleep in the prior sleep extension (PSE) condition and wakefulness with neurobehavioral testing in the prior sleep restriction (PSR) condition. Colored boxes denote 60-min neurobehavioral test bouts. Sleep inertia (SI) was quantified using sleepiness measured in the test bouts immediately after awakening marked with solid red boxes, relative to baseline sleepiness as averaged over the test bouts marked with open red boxes. Response to total sleep deprivation was assessed as the average of sleepiness measured in the test bouts marked with open blue boxes (from the PSE condition only). Open black boxes were not used for analyses. Participants underwent the schedule in this schematic three times—twice in the PSE condition and once in the PSR condition.

For each laboratory session, the test bouts at 10:00 right after awakening from baseline and recovery sleep were used to investigate sleepiness due to SI. The test bouts administered from 12:00 up to (but not including) 22:00 on the second day, before what would have been bedtime in the PSE condition (if not for the subsequent wake extension), were used to assess baseline sleepiness. The test bouts administered during the last 24 h of the 36-h total sleep deprivation (starting at 22:00)—a full circadian cycle of extended wakefulness—were used to assess vulnerability to sleep deprivation (for comparison with the effect of SI). See Figure 1.

### 2.3. Statistical Analyses

Nonlinear mixed-effects regression [34] was used to fit the effect of SI on sleepiness as observed in the KSS-1 of each of the test bouts administered at 10:00 right after awakening from sleep on the second and fourth days, relative to baseline sleepiness as observed in the KSS-1 of each of the test bouts from 12:00 up to (but not including) 22:00 on the second day (see Figure 1), in all three of the laboratory sessions in which every individual in the study participated. Based on previous research [14,15], it was assumed that the effect of SI decays exponentially as a function of time awake, and the regression equation was therefore specified as:

(1) $$y_{ij}(t_{ij})=I_{iks}e^{-t_{ij}/\tau }+B_{ikr}+\varepsilon_{ij},$$

where *y_ij_* denotes the sleepiness (KSS) score observed at time awake *t_ij_* for participant *i* in test bout *j*. The parameter *I_iks_* represents the estimated magnitude of SI for participant *i*, accounting for condition *k* (after baseline sleep in the PSE condition, after baseline sleep in the PSR condition, or after recovery sleep) and polysomnographically observed sleep stage from which awakening occurred *s* (stage 1, stage 2, slow wave sleep, or REM sleep). The parameter *τ* is the estimated time constant for the dissipation of the SI effect across time awake. The parameter *B_ikr_* represents the estimated baseline sleepiness for participant *i*, accounting for condition *k* (after baseline sleep in the PSE condition, after baseline sleep in the PSR condition, or after recovery sleep) assuming equivalence for after recovery sleep (when only one test bout was administered) to after baseline sleep in the PSE condition, and accounting for order effects *r* in the PSE condition (first session, second session preceded by PSE or PSR session, or third session preceded by PSE and PSR sessions or by PSR and PSE sessions) and in the PSR condition (first, second, or third session). Furthermore, *ε_ij_* denotes residual error assumed to be independent, normally distributed over participants and test bouts with mean 0 and variance *σ*^2^.

Independent, normally distributed random effects over participants *i* were placed on the SI magnitude *I_iks_* and the baseline sleepiness level *B_ikr_*, with means 0 and variances *ω*^2^ and *η* ^2^, respectively. Here, *ω* represents the standard deviation of systematic interindividual differences in the magnitude of the effect of SI on sleepiness. The stability of these interindividual differences was quantified with the intraclass correlation coefficient, ICC = *ω*^2^/(*ω*^2^ + *σ*^2^) [35], and determining the corresponding 95% confidence interval [36]. To verify the reliability of the interindividual differences estimate, the analysis was repeated with inclusion of the KSS-2 administered near the end of each test bout, accounting for any time-on-task effect associated with the test bout duration.

Additional analyses were performed for time awake and sleep stage at awakening. These analyses employed mixed-effects analysis of variance (ANOVA) with a normally distributed random effect with mean 0 and variance *ω*^2^ over participants on the intercept [30], assuming independent, normally distributed residuals with mean 0 and variance *σ*^2^. ICC values were calculated as described above. All analyses were performed in SAS 9.4 (SAS Institute, Cary, NC, USA).

## 3. Results

### 3.1. Sleep Stage at Awakening

For the 20 study participants and three laboratory sessions per participant, the sleep stage from which participants woke up and the time of final awakening could be accurately determined from polysomnography for all but nine baseline sleep periods (over seven different individuals) and nine recovery sleep periods (over eight different individuals). A total of 102 test bouts that occurred immediately after awakening from baseline or recovery sleep remained (i.e., about five assessments per participant on average), which could be used to estimate sleepiness due to SI. Table 1 shows a breakdown of the sleep stages from which participants woke up in the baseline and recovery sleep periods. There were no notable, systematic differences in sleep stage at awakening between individuals, as evidenced by the low ICC values in Table 1.

### 3.2. Time Awake at First KSS Administration

**Figure 2:**
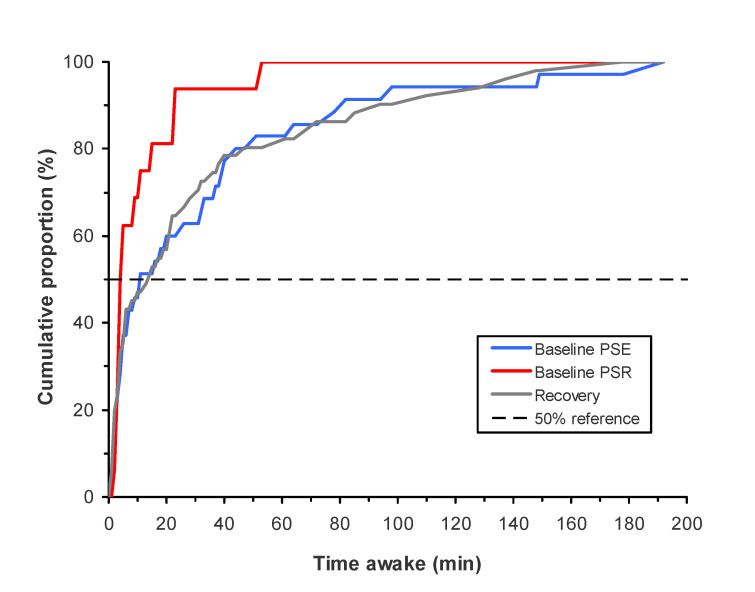
Cumulative distributions for how long participants had been awake at the time of the first Karolinska Sleepiness Scale (KSS-1) administration immediately after scheduled awakening. The curves show time awake since polysomnographically assessed final awakening in the 12-h baseline sleep opportunity in the PSE condition, the 6-h baseline sleep opportunity in the PSR condition, and the 12-h recovery sleep opportunity in either condition. The horizontal dashed line indicates the 50% cumulative proportion point.

Figure 2 shows cumulative distributions for time awake at the KSS-1 in the test bout right after awakening—i.e., for how long participants had been awake when the KSS-1 was administered immediately after the time of scheduled awakening. Given that awakening and the start of neurobehavioral testing were scheduled late in the morning at 10:00, participants often woke up earlier, but in this sample of healthy young adults the majority of polysomnographically assessed final awakenings still occurred less than 20 min before the first neurobehavioral test bout (Figure 2). 

The mean (±SD) for time awake at the KSS-1 in the test bout right after awakening was 32.1 ± 43.4 min after the 12-h baseline sleep period in the PSE condition; 11.2 ± 13.1 min after the 6-h baseline sleep period in the PSR condition; and 32.1 ± 43.1 min after the 12-h recovery sleep period. Compared to after baseline sleep in the PSE condition, time awake at this KSS-1 was significantly shorter after baseline sleep in the PSR condition (*F*_1,80_ = 7.27, *p* = 0.009), but there was no significant difference in time awake after recovery sleep (*F*_1,80_ < 0.01, *p* = 0.96). There were stable, systematic differences between participants in how long they had been awake when the KSS-1 right after awakening was administered, as evidenced by an ICC value of 0.545 (95% confidence interval: 0.355 to 0.742). These interindividual differences were accounted for in the statistical analysis of SI, as time awake was used as the independent variable in the statistical modeling of sleepiness due to SI; see below.

### 3.3. Magnitude of Sleep Inertia

**Figure 3:**
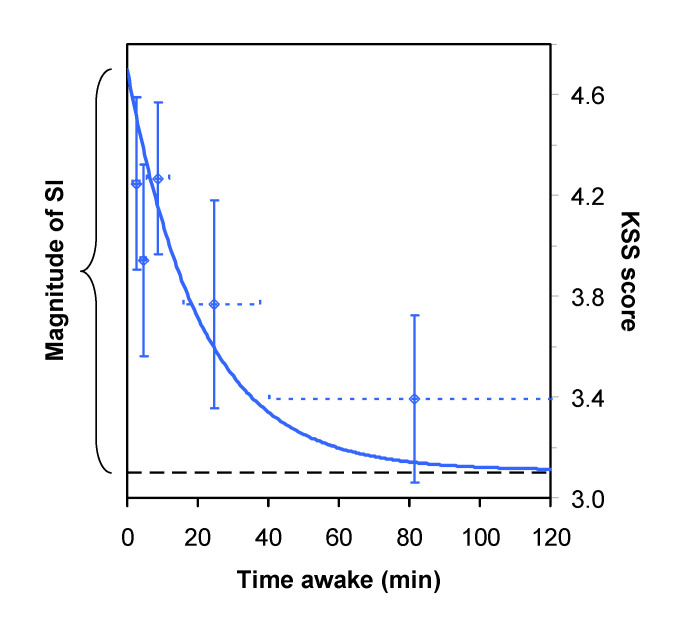
Nonlinear mixed-effects regression model for the KSS-1 administered immediately after scheduled awakening, as a function of time awake since polysomnographically assessed final awakening. The curve displays the group model for sleepiness (KSS score) from SI after baseline sleep in the first of the three laboratory sessions, in the PSE condition, assuming awakening from stage 1 sleep. The data points represent means of the overall set of observations for the KSS-1 administered immediately after scheduled awakening from baseline sleep, corrected for the model-estimated effects of laboratory session, prior sleep restriction, order effects, sleep stage upon awakening, and systematic interindividual differences in the magnitude of SI and baseline sleepiness—in groups of 10 observations (11 for the right-most data point) ordered by time awake. Vertical error bars indicate standard error of the mean; horizontal error bars indicate the time awake range for the observations captured in the mean. The horizontal dashed line denotes the baseline level of sleepiness to which the SI effect decays over time awake. The magnitude of the SI effect is defined as the maximum level of sleepiness immediately after polysomnographically assessed final awakening relative to the baseline level of sleepiness, as indicated by the curly bracket.

SI was well captured by our nonlinear mixed-effects regression model of sleepiness as a function of time awake, as shown in Figure 3 for the KSS-1 scores after awakening from stage 1 sleep at the end of the baseline sleep period in the PSE condition. Goodness-of-fit of the model was excellent as determined by likelihood ratio test (χ^2^_16_ = 259.2, *p* < 0.0001), and the model explained 61.1% of the variance in the data. The time constant for the exponential decay from peak sleepiness to baseline sleepiness was estimated to be *τ* = 20.2 min (SE: 11.5 min).

**Figure 4:**
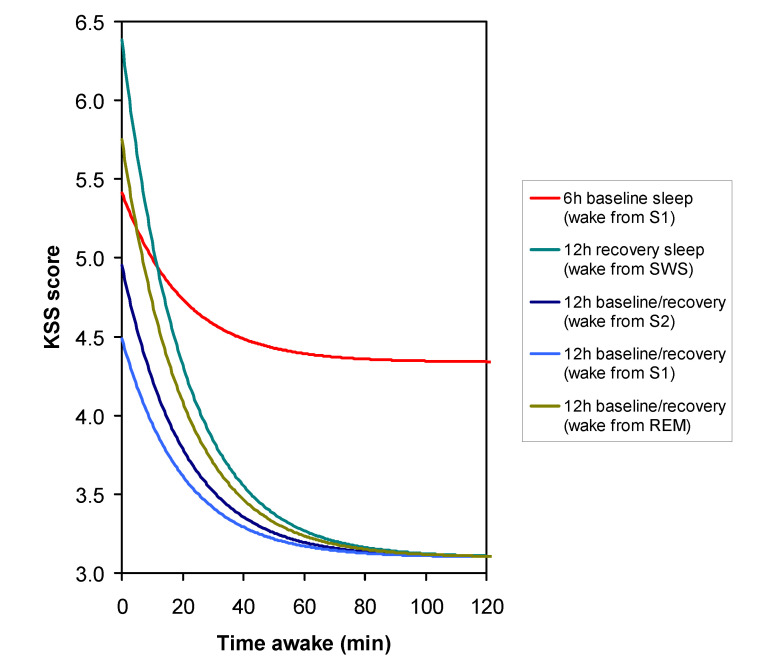
Nonlinear mixed-effects regression model for the KSS-1 administered immediately after scheduled awakening, as estimated for the first of the three laboratory sessions, comparing awakening from different stages of sleep and after prior sleep restriction. The curves display the group model for sleepiness (KSS score) from SI after the 12-h baseline sleep period in the PSE condition or the 12-h recovery sleep period, for which the trajectories overlap (except for awakening from slow wave sleep, which only occurred after recovery sleep), and after the 6-h baseline sleep period in the PSR condition (awakening from stage 1 sleep only). Note that the scale range on the ordinate is different from that in Figure 3.

Figure 4 compares the estimated sleepiness trajectories over time awake after awakening from the different sleep stages, where it was found that the SI profiles were the same after the 12-h recovery sleep period as after the 12-h baseline sleep period in the PSE condition (so the curves overlap, except for awakening from slow wave sleep, which only occurred after recovery sleep). Figure 4 also shows the estimated sleepiness trajectory after the 6-h baseline sleep period in the PSR condition, assuming awakening from stage 1 sleep. 

**Figure 5:**
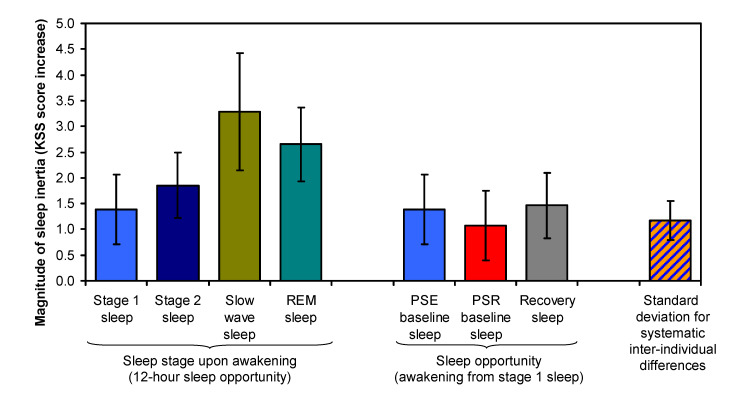
Magnitude of SI, estimated as the level of sleepiness immediately after awakening relative to the baseline level of sleepiness to which SI decays over time awake. The four bars on the left show the effect of SI on sleepiness (mean ± SE) after awakening from each of the different sleep stages in the 12-h sleep opportunities during the study (see Figure 1). The three bars in the middle compare the effect of SI on sleepiness (mean ± SE)—assuming awakening from stage 1 sleep—between the 12-h baseline sleep opportunity in the PSE condition (bar repeated from the left), the 6-h baseline sleep opportunity in the PSR condition, and the 12-h recovery sleep opportunity after 36 h of total sleep deprivation in either condition. For comparison, the bar on the right shows the standard deviation for systematic interindividual differences that persisted regardless of condition or sleep stage at awakening.

Figure 5 compares the estimated magnitudes of the effect of SI on sleepiness, where magnitude is defined as the maximum level of sleepiness immediately after awakening relative to the baseline level of sleepiness to which SI decays over time awake. As expected from the prior literature [21,22], the magnitude of SI was found to increase with the depth of non-REM sleep at awakening, from stage 1 to stage 2 to slow wave sleep (although the estimate for slow wave sleep was based on only three test bouts; see Table 1). Awakening from REM sleep was associated with an intermediate SI magnitude. The magnitude differences between the sleep stages were, however, not statistically significant (*F*_3,18_ = 2.01, *p* = 0.15). Furthermore, the magnitude of SI after the 12-h recovery sleep period was essentially identical to that after the 12-h baseline sleep period in the PSE condition (*t*_18_ = 0.16, *p* = 0.88). 

Paradoxically, the magnitude of SI after the 6-h baseline sleep period in the PSR condition was smaller than that after the 12-h baseline sleep period in the PSE condition, albeit not significantly (*t*_18_ = −0.51, *p* = 0.62). Yet, predictably, the baseline level of sleepiness after the restricted sleep in the PSR condition, to which the SI effect decayed over time awake, was significantly higher (*t*_18_ = 5.10, *p* < 0.001); see Figure 4. The KSS is highly compressed at the high end of the scale [29], such that higher scores are associated with progressively rapidly escalating levels of sleepiness. As such, the smaller magnitude of SI in the PSR condition could actually be associated with a subjectively more substantial impact of SI on sleepiness.

### 3.4. Trait Interindividual Differences

**Figure 6:**
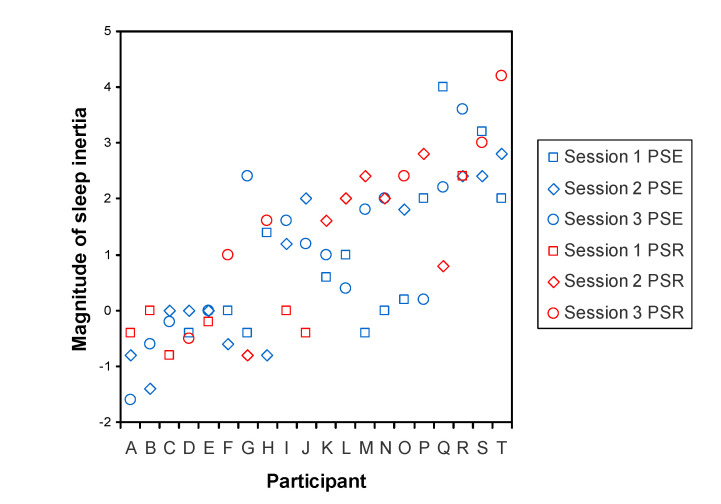
Systematic interindividual differences in the effect of SI on sleepiness following the baseline sleep period, illustrated by individual participants. Each participant underwent three separate laboratory sessions, the data of which are differentiated by different symbols. Randomized over the participants, one of the three laboratory sessions involved a PSR condition with a 6-h baseline sleep opportunity, whereas the other two laboratory sessions involved a PSE condition with a 12-h baseline sleep opportunity; these are differentiated by color. For this figure, the magnitude of the SI effect was determined as the sleepiness score on the KSS-1 in the test bout immediately after scheduled awakening, expressed relative to baseline sleepiness calculated as the mean of the sleepiness scores on the KSS-1 in the subsequent test bouts from 12:00 up to (but not including) 22:00 (see Figure 1). The abscissa shows the 20 individual participants, labeled A through T, ordered by their average magnitude of the SI effect, such that the least SI-susceptible individuals are on the left and the most SI-susceptible individuals are on the right. The figure shows that individuals differed substantially in the magnitude of the effect of SI on sleepiness, while the effect was relatively consistent within individuals, reflecting stable interindividual differences in the magnitude of SI.

Importantly, Figure 5 also displays the considerable size of interindividual differences in the magnitude of SI, shown as the standard deviation of systematic interindividual differences that persisted between the PSE and PSR conditions, after total sleep deprivation, and across the different sleep stages at awakening. This standard deviation was statistically significant (*t*_18_ = 3.15, *p* = 0.006) and estimated to be *ω* = 1.17 units (SE: 0.37 units) on the KSS. The effect of SI on sleepiness was stable within individuals, as evidenced by an ICC value of 0.511 (95% confidence interval: 0.363 to 0.698; variance components: *ω*^2^ = 1.373, *σ*^2^ = 1.315). The ICC was statistically significant (*F*_19,334_ = 22.91, *p* < 0.001). To illustrate these results, Figure 6 provides a graphical representation of the systematic differences between individuals in the effect of SI on sleepiness as observed after the baseline sleep opportunity.

When the analysis was repeated with inclusion of the KSS-2 of each test bout, accounting for the time-on-task effect associated with the test bout duration (estimated to be 0.49 ± 0.10 units on the KSS), the results were similar. In this secondary analysis, it was found that *ω* = 1.03 ± 0.38 (*t*_18_ = 2.70, *p* = 0.015) and ICC = 0.406 (*F*_19,689_ = 29.73, *p* < 0.001). Although the similarity of these additional results is not surprising, as the effect of SI on the KSS-2 (administered almost an hour after the first) was very small even in the first test bout after awakening, it provides confidence in the reliability of our finding of considerable, stable interindividual differences in the magnitude of SI.

**Figure 7:**
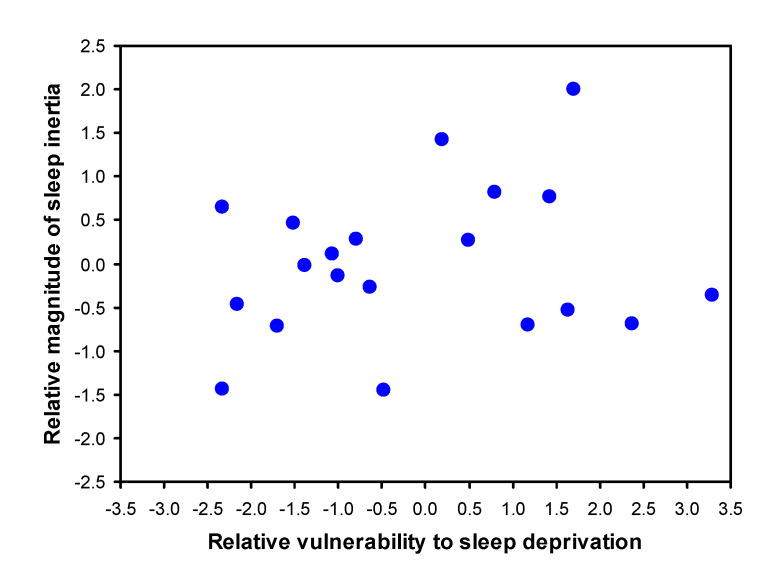
Comparison of interindividual differences in susceptibility to sleepiness due to SI and vulnerability to sleepiness during total sleep deprivation. The ordinate shows individual estimates of the magnitude of SI, relative to baseline sleepiness, expressed as difference from the group mean. The abscissa shows individual estimates of the effect of sleep deprivation, relative to baseline sleepiness, expressed as difference from the group mean. Both are in units on the KSS. Notice that the variability between individuals in the effect of SI on sleepiness is only somewhat smaller than the variability between individuals in the impact of sleep deprivation on sleepiness—however, the two effects are not significantly related within individuals.

To get a sense of the scale of the interindividual differences in the magnitude of SI, estimates of the effect of SI on sleepiness were compared to estimates of the effect of total sleep deprivation on sleepiness in the same individuals. Interindividual differences in vulnerability to sleepiness due to sleep deprivation in this data set were previously shown to be both substantial and trait-like [28]. For the present purpose, individual vulnerability to sleepiness due to sleep deprivation was quantified as the mean of the KSS-1 scores in each of the test bouts from 22:00 on the second day until 20:00 on the third day (i.e., during the last 24 h of the 36-h total sleep deprivation period). This was expressed relative to baseline sleepiness, calculated as the mean of the KSS-1 scores in each of the test bouts from 12:00 up to (but not including) 22:00 on the second day (see Figure 1). The result was averaged over the two PSE condition laboratory sessions (i.e., when participants were well rested beforehand). Individual susceptibility to sleepiness due to SI was quantified using the empirical Bayes estimates [35] for the random effect on the SI magnitude in our nonlinear mixed-effects regression model for SI. Figure 7 shows the comparison between individuals’ susceptibility to SI and their vulnerability to sleep deprivation in the form of a scatter plot. The range of the differences between individuals was largest for vulnerability to sleep deprivation, but not by much. The two effects were not strongly related, however, as corroborated by a small and nonsignificant rank-order correlation (*ρ* = 0.205, *p* = 0.40).

## 4. Discussion

Under highly controlled laboratory conditions, we investigated whether subjective sleepiness due to SI was (a) substantially different between individuals; (b) highly stable within individuals; and (c) robust to experimental manipulation of sleep/wake history. We observed substantial interindividual differences in sleepiness due to SI, which were stable across two laboratory sessions with PSE and a laboratory session with PSR (Figure 6) and persisted after recovery from 36 h of total sleep deprivation. Meeting the above criteria (a) through (c), our results provide the first systematic evidence that interindividual differences in subjective sleepiness due to SI constitute a trait or phenotype. 

The standard deviation of the systematic interindividual differences in the magnitude of SI was approximately the same size as the estimated mean SI effect after awakening from stage 1 sleep (Figure 5), indicating that the full range of the interindividual differences (which can be seen in Figure 7) was substantial. Given that estimates of interindividual variability depend on the sample studied [30] and considering that we studied a relatively homogenous sample of healthy young adults, it is possible that the range of interindividual differences in susceptibility to SI is even greater in the general population. Yet, even in the present sample, the range of interindividual differences in the magnitude of SI was comparable to the considerable interindividual differences we observed in vulnerability to sleep deprivation, which has already previously been shown to be a trait [28]. Perhaps surprisingly, though, the two traits did not appear to be closely related within individuals (Figure 7). This does not necessarily reveal whether or not the two phenomena—susceptibility to SI and vulnerability to sleep deprivation—may have linked underlying mechanisms [25] or could be predicted by the same gene polymorphisms [37]. However, it does rule out the possibility that the observed interindividual differences are merely a reflection of idiosyncratic differences in how individuals interpret and use the Likert-type scale of the KSS.

Based on earlier research [14,15], we assumed an exponential decay of the SI effect over time awake, which was corroborated by the excellent goodness-of-fit of our nonlinear mixed-effects regression model to the data (Figure 3), explaining 61.1% of the variance. Our estimate of the time constant for the decay of the SI effect across time awake (i.e., the time it takes for SI to be reduced to 36.8% of the initial effect) was 20.2 min, which is similar to what has been found previously [14]. The time constant estimate implies that 30 min of time passing since awakening results in a dissipation of SI to less than 25% of the initial effect, with only about 5% of the effect left after an hour. These numbers are consistent with estimates from the earlier research [14,15]. 

In the majority of cases, the KSS-1 assessment after each sleep period (at 10:00) occurred within 20 min of the polysomnographically assessed time of final awakening. Even though the PSE condition offered more than adequate rest prior to laboratory baseline sleep [27,28,38], it is not surprising that participants often slept late into the morning, because our sample consisted of healthy young adults who may need as much as 9 h of sleep per night or more [39]. Even so, a wide range was observed for time awake prior to the first test bout, especially for the 12-h baseline and recovery sleep periods (Figure 2). This variability in the relative test bout timing was harnessed effectively with our nonlinear mixed-effect modeling approach [34] to estimate the SI effect—not only at the time of neurobehavioral testing but also, importantly, at the polysomnographically assessed time of awakening (Figure 3). 

Approximately three quarters of all final awakenings occurred from stage 1 or stage 2 sleep, and about a quarter from REM sleep, which is consistent with the distribution of sleep stages toward the end of a typical nocturnal sleep period [40]. Only for the recovery sleep after 36 h of total sleep deprivation did we observe a few instances of awakening from slow wave sleep (Table 1). Given the increased pressure for slow waves in the sleep EEG after sleep deprivation [41] and the previously described sleep stage dynamics of long sleep periods [42], this is consistent with expectation. Interestingly, no systematic differences between individuals were observed in the sleep stage of awakening. Yet, the sleep stage from which participants woke up showed a systematic influence on SI (Figure 4), as has been reported in earlier studies [16,21,22]. Specifically, the deeper the stage of non-REM sleep (from stage 1 to stage 2 to slow wave sleep), the greater the magnitude of SI (Figure 5). The magnitude of SI following awakening from REM sleep was between that from stage 2 and slow wave sleep, which is higher than expected. However, our sample size was not large enough to differentiate the influence of the different sleep stages with statistical significance—in contrast with our main investigation of trait interindividual differences in the magnitude of SI, for which we had ample statistical power. 

It should be noted that the timing of scheduled awakenings was held constant throughout this study, so that circadian effects on the magnitude of SI [16,17] were not a confounding factor, but could also not be investigated here. Additionally, it remains to be investigated whether interindividual differences in the effect of SI on subjective sleepiness may translate to similar interindividual differences in the impact of SI on other neurobehavioral measures, such as performance on cognitive tasks or safety in operational settings. The literature is inconclusive in this regard [11,13]. Future studies of SI designed to further assess trait interindividual differences should be able to provide insight regarding this important issue.

## 5. Conclusions

In this study, we showed that interindividual differences in the magnitude of subjective sleepiness as a consequence of SI range from negligible to substantial and are stable and robust within individuals, thereby constituting a human trait or phenotype. This finding may have profound implications in situations where individuals are relied upon to be alert and perform well soon after being awakened. This includes on-call and emergency response operations [43,44], workplace napping and split duty schedules [8,45], and—potentially relevant in the near future—semiautomated driving scenarios [46]. All of these circumstances may put some individuals at much greater risk from SI than others, depending on their SI phenotype. Knowing in advance who is most at risk from SI, e.g., based on earlier observation of a person’s SI response, would be helpful to target SI countermeasures at those who may need them most. Using the KSS or other self-report sleepiness scale would provide a quick and effective assessment of a person’s subjective SI in operational settings.

A variety of SI countermeasures has been explored [47], including restricting sleep duration in an attempt to avoid slow wave sleep [48], consuming caffeine immediately after awakening [24], “caffeine-napping” (i.e., consuming caffeine immediately before taking a nap) [49], exposure to blue light [50] or red light [51] or simulated dawn [52], auditory stimulation [53], exercise [54], implementation of an advance wake-up call program [55], and delaying safety-critical tasks until SI has dissipated (e.g., by engaging in small talk before turning to critical decision making) [56]. Insofar as these countermeasures have been systematically evaluated, those that seek to accelerate the dissipation of SI have largely yielded mixed evidence of effectiveness. In light of trait interindividual differences in the magnitude of SI, this may be inevitable—after all, only those who are most impacted by SI would stand to benefit significantly from mitigating its effects. Further studies that account for the substantial interindividual variability in SI are needed to elucidate which SI countermeasures may be most effective and for whom.

## Figures and Tables

**Table 1:** Breakdown of polysomnographically assessed sleep stages from which participants woke up in the baseline and recovery sleep periods. The table shows percentages (and counts) of the different sleep stages for the distinct types of sleep periods in the study, as well as intraclass correlation coefficient (ICC) values (and their 95% confidence intervals) for systematic interindividual differences.

Sleep Stage at Awakening	12-h Baseline (PSE Condition)	6-h Baseline (PSR Condition)	12-h Recovery	ICC
Stage 1 sleep	28.6% (10)	25.0% (4)	41.2% (21)	0.024 (0.000–0.207)
Stage 2 sleep	48.6% (17)	56.3% (9)	29.4% (15)	0.116 (0.000–0.333)
Slow wave sleep	0% (0)	0% (0)	5.9% (3)	0.000 (0.000–0.171)
REM sleep	22.8% (8)	18.7% (3)	23.5% (12)	0.113 (0.000–0.329)

## Data Availability

Upon reasonable request from H.P.A.V.D. the data can be shared with researchers.
